# Nanoscale High-Tc YBCO/GaN Super-Schottky Diode

**DOI:** 10.1038/s41598-018-23882-6

**Published:** 2018-04-04

**Authors:** Dmitry Panna, Krishna Balasubramanian, Shlomi Bouscher, Yujia Wang, Pu Yu, Xi Chen, Alex Hayat

**Affiliations:** 1 Department of Electrical Engineering, Technion, Haifa, 32000 Israel; 2 State Key Laboratory of Low Dimensional Quantum Physics and Department of Physics, Tsinghua University, Beijing, 100084 China

## Abstract

We demonstrate a high-temperature nanoscale super-Schottky diode based on a superconducting tunnel junction of pulsed-laser-deposited YBCO on GaN thin films. A buffer-free direct growth of nanoscale YBCO thin films on heavily doped GaN was performed to realize a direct high-T_c_ superconductor-semiconductor junction. The junction shows strongly non-linear I-V characteristics, which have practical applications as a low-voltage super-Schottky diode for microwave mixing and detection. The V-shaped differential conductance spectra observed across the junction are characteristic of the *c*-axis tunneling into a cuprate superconductor with a certain disorder level. This implementation of the super-Schottky diode, supported by the buffer-free direct growth of nanoscale high-T_c_ thin films on semiconductors, paves the way for practical large-scale fabrication and integration of high-T_c_-superconductor devices in future technologies.

## Introduction

The phenomenon of superconductivity and its observation in a wide range of temperatures have captured the curiosity of scientists and engineers for several decades^1^. In addition to opening several avenues in fundamental science research, superconductors and their combinations with other materials have numerous practical applications. Ranging from radio astronomy^2^ and nuclear research^3^ to medical imaging^4^ and transportation, devices with conventional low-critical-temperature (low-T_c_) superconductors are currently being commercially employed in quantum computing circuitry^5^, single-photon detection^6^, ultra-sensitive magnetic flux detection^7^, and for generating the universal standard for voltage^8^. Direct contact between superconductors and semiconductors opens a wide range of exciting pathways leading to enhanced photoemission from semiconductors^9^, enhanced two-photon gain^10^ and efficient sources of entangled photons^11^ for quantum information processing, Josephson junctions^12^ and many more. A super-Schottky diode is a superconductor-semiconductor tunnel junction with very low turn-on voltage, extraordinarily low noise performance^13^ and a high degree of non-linearity, which enable high sensitivity microwave and video signal detection as well as microwave mixing^14^. Implementing such devices with high-T_c_ superconductors with an order-of-magnitude higher operating temperature^15,16^ such as YBa_2_Cu_3_O_7_ (YBCO)^17^, could pave the way for widespread use of superconductor-based technologies. Moreover, direct contact between high-T_c_ materials and semiconductors can enable more practical high-temperature superconducting optoelectronics, so far studied only with low-T_c_ materials. Despite decades of intensive research on high-T_c_ superconductor material physics, devices based on high-T_c_ superconductors directly grown on technologically important semiconductors, such as the III-V material family, have not been demonstrated before, due to the challenging high-T_c_ thin-film growth conditions^18^. Epitaxial YBCO films with excellent quality were obtained using pulsed laser deposition (PLD) on insulating substrates such as Strontium Titanate (SrTiO_3_), Lanthanum Aluminate (LaAlO_3_)^19^ and their doped films^20,21^. Several works have demonstrated PLD growth of YBCO thin films on insulators such as Zirconia and MgO as buffer layers on semiconducting materials such as Si^22^ and GaN^23^. However, due to the extremely short coherence lengths in these high-T_c_ superconductors and the large potential barrier presented by the insulating buffer, proximity of a superconductor wavefunction to the semiconductor is virtually impossible through the buffer layers. Recently, an alternative technique of mechanical bonding was employed to implement a high-T_c_ superconductor-semiconductor junction with a bulk BSCCO crystal bonded to a semiconductor^24^. Nevertheless, the large size of such bulk crystal-based devices prevents the miniaturization of superconductor-semiconductor technology. Development of direct-contact thin film high-T_c_ superconductor-semiconductor devices can enable large-scale integration of superconductor electronic and optoelectronic devices.

Here we demonstrate direct PLD growth of a nanoscale thin film YBCO on a III-V semiconductor – GaN, and realize a high-T_c_ super-Schottky diode. We show that GaN is sufficiently robust to withstand the high temperatures required for YBCO thin film growth without significantly affecting its quality and stability^25^. Moreover, GaN is a technologically important material with applications in blue light-emitting diodes, high-power electronic devices and amplifiers^23^. We performed electrical transport measurements on the fabricated YBCO-GaN junction, showing strong nonlinearity for bias voltages within the superconductor energy gap Δ, which is a primary requirement for super-Schottky diode applications. We describe the growth, processing and characterization of the YBCO/GaN devices and finally, implement a theoretical model to extract the junction interface quality.

## Methods

**Figure 1:**
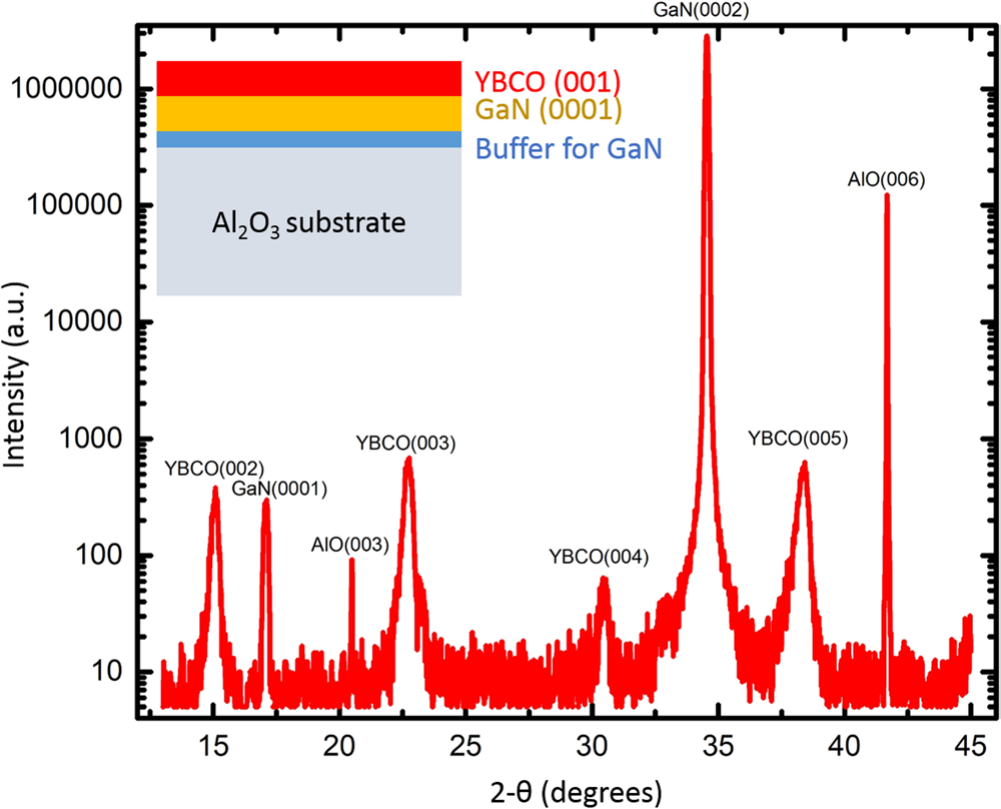
A full range thin film X-Ray diffraction measurement on the heterostructure. The peaks are indexed with the material system and the orientation. (001) oriented YBCO film can be identified along with the (0001) oriented GaN film. The inset shows the device structure.

For our devices, 2 *µ*m thick heavily doped n-type GaN film (n ~ 5∙10^18^ cm^−3^) was initially grown on buffered Al_2_O_3_ (001) substrate. The degenerate doping of GaN enables a good contact with the superconductor and prevents carrier freeze-out, thus enabling low-temperature super-Schottky diode operation. The wafer was cleaned using acetone and isopropanol, followed by annealing in air at 900 °C for 1 hour. Then the native oxide removal has been performed in an ultra-high vacuum chamber at 850 °C for half an hour. The YBa_2_Cu_3_O_7−x_ layer was subsequently grown using a customized PLD system (TSST). A KrF excimer laser (248 nm) with pulse fluence of 1.4 J/cm^2^ and repetition rate of 3 Hz was used. During the deposition, the sample was kept at a temperature of 650 °C and under oxygen pressure of 0.19 mbar. After the growth, the sample was cooled down in oxygen atmosphere of 100 mbar to avoid the formation of oxygen vacancies. Finally, for improving superconducting properties, the YBCO/GaN/Al_2_O_3_ structure was annealed in ozone at 300 °C for 1 hour. A schematic representation of the material stack and the representing X-Ray diffraction scan after the growth is shown in Fig. 1. Films with different thickness were grown and two films with thickness of 40 nm and 80 nm labeled as Y40 and Y80 were used for further measurements.

## Results and Discussion

**Figure 2:**
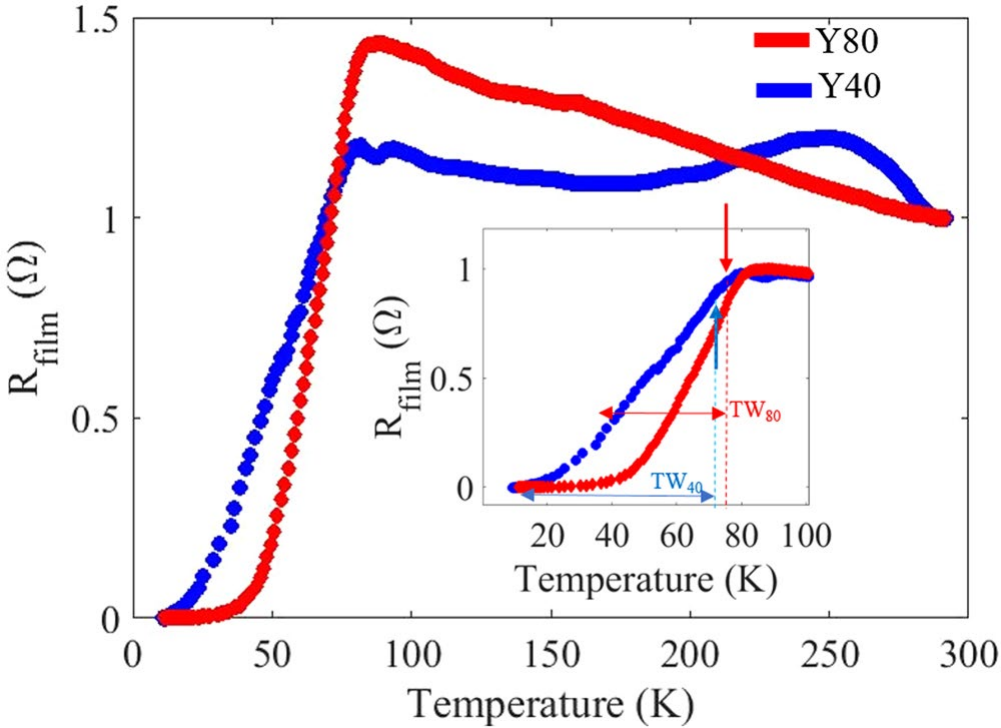
Four-probe resistance measurement as a function of temperature for two YBCO films. The 40 nm and 80 nm films have different resistance roll-off rates but the superconducting transition temperature was about 80 K for both films.

The X-ray diffraction measurement shows multiple peaks corresponding to the YBCO film orientation, the GaN film and the sapphire substrate. Several higher-order peaks of these materials are also seen and marked accordingly in Fig. 1. The sharp (0002) peak of GaN shows the high crystalline nature of the *c*-axis GaN semiconducting film. Similarly, sharp peaks corresponding to (001) family of YBCO planes are seen indicating good crystalline quality. Absence of any other orientations other than (001) family of the YBCO in the full range measurement also shows that the YBCO film is crystalline with strong epitaxial relationship with the underlying (0001) GaN film. Close match of the lattice spacing and the 2-theta values of the YBCO thin film peaks with those of the bulk indicate relatively strain-free growth with pure stoichiometry. Assured of the quality, the films were tested for the superconducting transition temperature. Figure 2 shows four-probe resistance measurements as a function of temperature conducted on YBCO films with both thicknesses.

Both films exhibit superconducting transition onset (*T*_*c*_) well above the liquid nitrogen temperature. However, it should be noted that the *T*_*c*_ and the transition width (TW) have large deviations from the best reported bulk YBCO values^26^. The depression in the *T*_*c*_ for thin films has been previously reported and were attributed to several reasons such as strain, proximity effects, strong localization effects in disordered systems, and modified boundary conditions for the order parameter calculations in the Ginzburg-Landau free energy expressions^27^. Although both films have a slightly lower *T*_*c*_ than optimally doped YBCO, the shift in *T*_*c*_ is similar for the films with different thicknesses. This shows that the dominant source of the *T*_*c*_ suppression does not depend on the film thickness. Since total strain energy and proximity effects vary significantly with thickness, the most likely source of *T*_*c*_ depression can be attributed to defect density close to the GaN/YBCO interface. The most striking difference between the films is the width of the superconducting transition. As the film thickness is increased, the dislocation density^28^ is expected reduce due to bending^29^. In the case of YBCO grown on MgO, it has been found to reduce by an order of magnitude for a thickness of 100 nm^30^. Thus, 40 nm film and 80 nm film on GaN are expected to have different defect density, averaged over thickness. In addition to the defects, elemental diffusion from the bottom layers should also be considered. In the case of Si, it prevented direct integration with superconductor and required a buffer layer^31,32^. In our work, Ga from the GaN layer could diffuse during growth. Since, both films in our case were grown under the same temperature, meaning the diffusion length being the same, the effect is expected to be more pronounced in the thinner 40 nm films in contrast to thicker one, leading to degraded (longer roll-off) transition characteristics in the thinner films. We note that other conclusions as will be discussed further also support the hypothesis.

**Figure 3:**
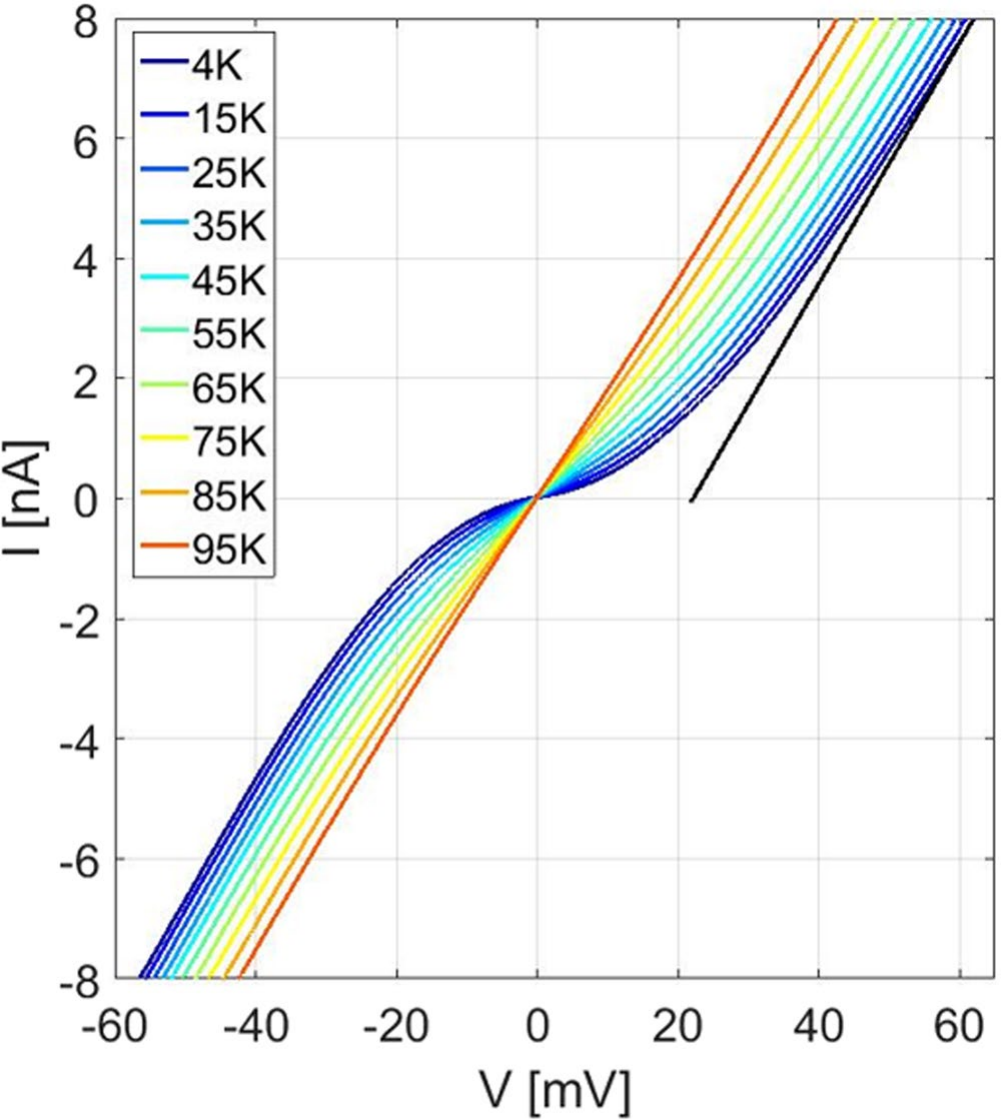
Super-Schottky diode characteristics of the 80 nm YBCO film on GaN at various temperatures. A linear IV behavior is seen at the temperatures above the superconducting transition. A clearly non-linear behavior at lower temperatures and an excess voltage close to the superconducting gap are noted. The black line is the linear fit at higher bias voltages, an x-axis intersection indicates an excess voltage of the junction.

We proceed to electrical transport measurement across the interface of the nanoscale high-T_c_ super-Schottky diode. A portion of the grown YBCO film was wet etched using 1% phosphoric acid solution and contacts on both the YBCO and the GaN films were made. The junction current-voltage (IV) characteristic is presented in Fig. 3 for various temperatures. The junction exhibits non-linear behavior at temperatures below the superconducting transition temperature of the YBCO film. The excess voltage^24^ could be extrapolated from a linear fit to the portion of the IV curve at higher voltages. It is this non-linearity in the IV below the superconducting gap that is employed for the implementation of the super-Schottky diode^14^. However, in contrast to the standard Schottky diode behavior, symmetric IV curves with *I*(−*V*) = −*I*(*V*), close to zero bias, are expected for super-Schottky diodes^33^ - due to the superconducting gap being the source of the device nonlinearity. For the super-Schottky diode, to improve the superconductor-semiconductor interface quality, degenerate doping of the semiconductor is intentionally employed. Therefore, the non-superconducting Schottky effect is significantly reduced, leading to almost symmetric forward and reverse characteristics. The tunneling conductance is further studied below the superconducting gap to understand the diode behavior in the highly non-linear region.

**Figure 4:**
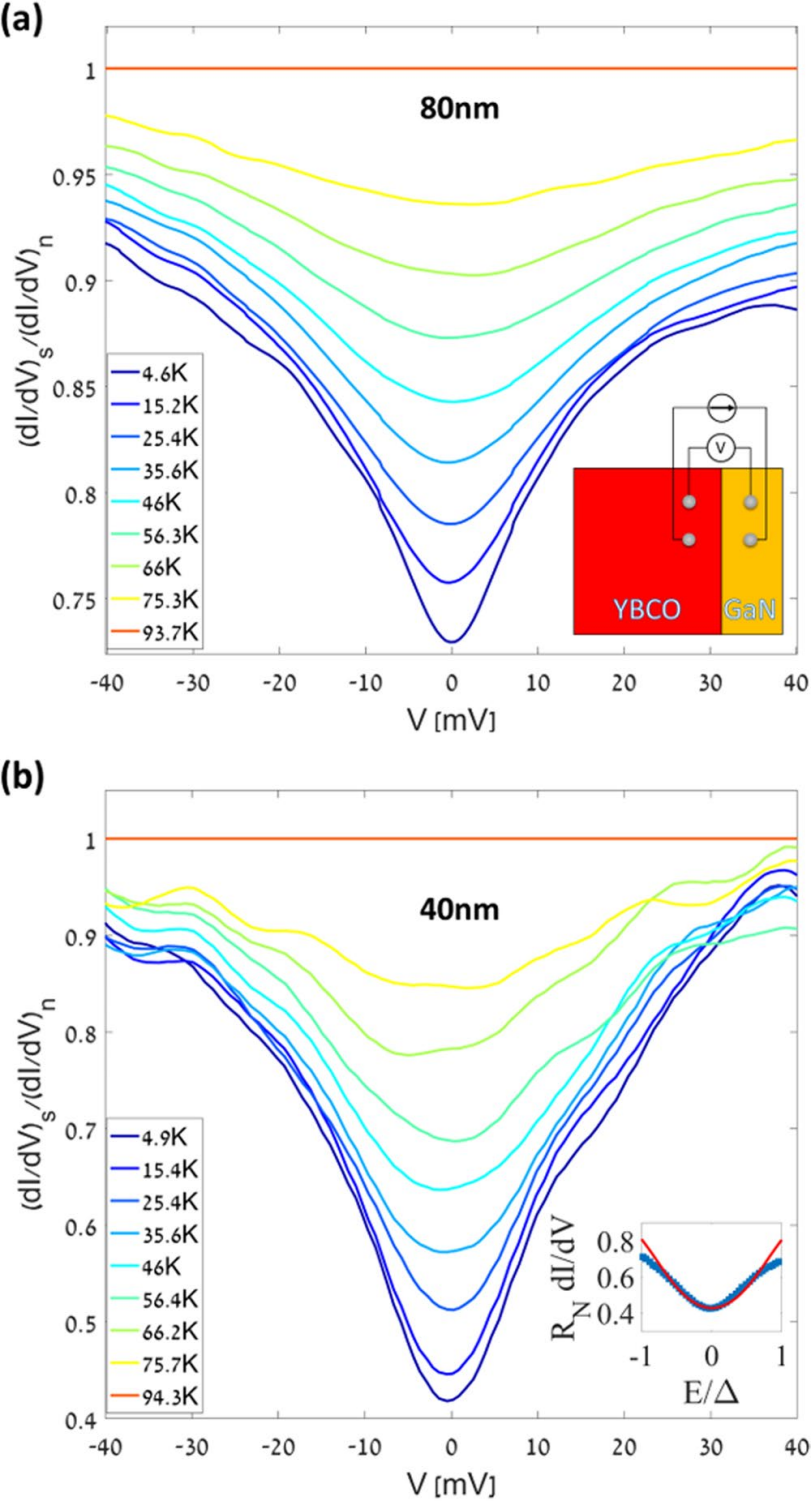
(**a**) Normalized differential conductance spectra of Y80 for various temperatures. Inset is the device sketch and a 4-probe measurement scheme. (**b**) Corresponding plot for Y40. A clear V shaped conductance spectra are observed due to the *c*-axis tunneling from *d*-wave superconductors with some disorder. Inset depicts tunelling characteristics of Y40 (blue) and its theoretical modeling (red).

Transport across a semiconductor-normal interface can be calculated using approaches based on the Blonder-Tinkham-Klapwijk model^34^. The quasi-particle tunneling rate across the junction depends on both superconductor and semiconductor density of states, Fermi-Dirac distributions and tunneling matrix elements, which are closely related to the interface quality. Consequently, transport measurements across such interfaces are considered as the most direct probe of the interface quality, absolute value and the symmetry of the superconductor gap, and the density of quasi-particle states of the superconductor^35,36^. Measurements on YBCO junctions alone (without semiconductors) have been carried out through various means^37^. In our experiments, we investigate planar tunneling through the *c*-axis of the (001) oriented YBCO grown directly on GaN. Differential conductivity measurements were performed using a four-probe configuration as shown in the inset of Fig. 4a. The differential conductance dI/dV of the junction was measured by supplying a small AC component super-imposed over a variable DC offset. A phase-sensitive detection using a lock-in amplifier enables detection of the small-signal device response. The measured differential conductance spectra for $T < {T}_{c}$ were normalized by the normal state differential conductance at $T > {T}_{c}$. The normalized differential conductance spectra of Y80 and Y40 are presented in Fig. 4a,b respectively. In both films a distinct V shaped conductance dip is seen, which disappears as the temperature is raised above *T*_*c*_. However, typically the normalized conductance displays peaks close the energy gap edge, indicative of long range coherence^38^, which do not appear in our measurements. In superconductors with disorder, localization of Cooper pairs was predicted with a loss of phase coherence, subsequently leading to strongly reduced coherence peak height in the conductance plots^39^. Such effects have been experimentally observed and gapped conductance plots with flattened peaks have also been shown in superconductors with disorder^40^. In both our films, Y80 and Y40, lower transition temperature and slower roll-off to zero resistance are indicative of high disorder density^41^. Hence, the flattened peaks with a gap in the tunneling conductance are attributed to the disorder in the system leading to loss of long-range phase coherence. While the V-shaped tunneling characteristics is typical of *c*-axis tunneling in YBCO^42^ with a certain level of disorder, on a closer look the characteristics of both the films are different in the ratio of the zero-bias conductivity to the normal state conductance.

To understand the origin of these differences, the tunneling characteristics were compared with the theoretical model developed by Kashiwaya *et al*.^43^ with modifications to account for broadening (ζ). The normal junction conductivity is expressed as ${\sigma }_{N}\equiv \frac{4\lambda }{{(1+\lambda )}^{2}+4{Z}^{2}}$, where λ is the wavevector mismatch between the semiconductor and the superconductor quasiparticles, and interface impedance parameter $Z=\frac{{Z}_{0}}{\cos ({\theta }_{N})}$, where ${Z}_{0}=\frac{mH}{{\hslash }^{2}{k}_{FN}}$ is the measure of the interface impedance, $\hslash$ is the reduced Planks constant and $m$ is the mass of the electron. The normalized conductance at an angle *θ*_*N*_ is

1 $${\sigma }_{R}=\frac{1+{\sigma }_{N}{|{{\rm{\Gamma }}}_{+}|}^{2}+({\sigma }_{N}-1){|{{\rm{\Gamma }}}_{+}{{\rm{\Gamma }}}_{-}|}^{2}}{|1+({\sigma }_{N}-1){{\rm{\Gamma }}}_{+}{{\rm{\Gamma }}}_{-}\exp (i[{\phi }_{-}-{\phi }_{+}])|}$$

where ${{\rm{\Gamma }}}_{\pm }=\frac{E-{{\rm{\Omega }}}_{\pm }}{{{\rm{\Delta }}}_{\pm }}$, ${{\rm{\Omega }}}_{\pm }=\sqrt{{E}^{2}-{|{{\rm{\Delta }}}_{\pm }|}^{2}}$, ${{\rm{\Delta }}}_{\pm }$ is the pair potential of the electron-like and hole-like quasi-particles and ${\phi }_{\pm }$ are the phases of the effective pair potentials. We calculated the tunneling spectra which agrees well with our measurements (Fig. 4b. inset).

An additional broadening due to scattering was included in the calculations by adding an imaginary term to the energy^24^. The theoretically evaluated tunneling conductivity fits well with the experimental values obtained. An interface impedance value Z of 1.5 was obtained for Y80 and 1.8 for Y40. For Y80, best fits were obtained with a pair potential of 18.2 mV and broadening factor ζ = 11 meV. While the fits to the experimental values of Y40 were obtained for the pair potential of Δ = 17.6 mV with a barrier strength parameter of ζ = 13 meV. Evidently, the observed tunneling conductance difference between Y80 and Y40 in Fig. 4 is a manifestation of smaller gap in the density of states of Y40. In addition, the larger broadening parameter, (which was modeled according to Dynes broadening^44^ obtained from the fits to the Y40 also concurs with the observation made previously from the Fig. 2 on the larger transition widths. The defect density varies from the interface through the thickness of the film due to dislocation bending and annihilations as discussed before^29,30^. Diffusion from the bottom layers also has a more significant effect on the thinner 40 nm film compared to the 80 nm one. Hence, even as the growth conditions of both the films were identical, the Y40 displays a smaller gap and larger broadening due to inhomogeneity effects than the thicker Y80.

## Conclusions

In conclusion, we have demonstrated a super-Schottky tunnel diode with YBCO nanoscale films on GaN. The device exhibits strong non-linearity at sub-gap excitations, proving its suitability as an efficient device architecture for the super-Schottky diode. The buffer-free growth and degenerate doping enables a direct contact between the semiconductor and the superconductor. The theoretical modeling of the experimentally observed tunneling characteristics points to lower interface impedance parameters, reinforcing the usefulness of direct semiconductor-superconductor junction. This integration of the two layers opens pathways for future large-scale manufacturing of high-T_c_ superconductor-semiconductor devices and their integration with other components on a single platform.
